# Supplementary material for: Extreme sensitivity to ultraviolet light in the fungal pathogen causing white-nose syndrome of bats
Source: Nat Commun. 2018 Jan 2;9:35. doi: 10.1038/s41467-017-02441-z (PMC5750222; doi:10.1038/s41467-017-02441-z)
Supplement: Supplementary file 1 — Supplementary Information [file 41467_2017_2441_MOESM1_ESM.pdf]

Supplementary Methods

Genome annotation and functional characterization

Repetitive elements in the *P. destructans* assembly were identified using RepeatModeler v1.08[1] was and were subsequently soft-masked using RepeatMasker v4.05 [2]prior to downstream genome annotation. For *P. destructans*, RNA-sequencing data was leveraged to improve gene prediction accuracy, including RNA-sequencing data from laboratory culture conditions (SRP001346) as well as from WNS-infected bats (SRP055976). The Trinity v2.1.1 software package[3] was used to assemble RNA-sequencing data *de novo* into transcripts and HISAT v2.0.0[4] was used to align RNA-sequencing reads to the *P. destructans* assembly. Trinity transcript assemblies were passed to PASA v2.0.2[5] to identify high quality transcript assemblies derived from the RNA-sequencing data. The aligned RNA-sequencing data was then used to train the *ab initio* gene prediction programs AUGUSTUS v3.1.0[6] and GeneMark-ET v4.32[7] via the BRAKER1 v1.8 pipeline[8]. Protein alignments from the UniProt/SwissProt database to the *P. destructans* assembly were generated using exonerate[9] and Trinity/PASA transcripts were aligned to the genome using GMAP[10]. Genes encoding tRNA's were identified using tRNAscan-SE v1.23[11]. Gene prediction for the 6 non-pathogenic *Pseudogymnoascus* species was conducted similarly, with some changes to the pipeline because the RNA-sequencing data obtained from the Ion Torrent PGM did not contain enough reads to adequately run the PASA pipeline. Thus, RNA-seq data was converted to transcripts using genome guided Trinity, AUGUSTUS was trained using BUSCO[12], and GeneMark-ES v4.32 was used for *ab initio* gene prediction. Additionally, ESTs downloaded from JGI Mycososm (http://genome.jgi.doe.gov/programs/fungi/index.jsf) for closely related species (Leotiomycetes) were used in addition to the Trinity transcripts for generation of transcript alignments using GMAP. Finally, EVidenceModeler v1.1.1[5] was used to combine data from protein alignments, transcript alignments, and *ab initio* predictions to construct high quality evidence based gene models.

The resulting annotation was converted to GenBank flat file format using the Genome Annotation Generator (GAG)(https://github.com/genomeannotation/GAG) followed by tbl2asn v25.3 (https://www.ncbi.nlm.nih.gov/genbank/tbl2asn2/). Gene models that resulted in tbl2asn errors were manually fixed or removed. Functional annotation was performed using HMMER v3.1b2.1 (http://hmmer.org/) and BLAST v2.2.31+[13] searches using several curated databases: pfam-A database[14], InterProScan5 database[15], MEROPS protease database[16], dbCAN CAZyme database[17], UniProt/SwissProt database[18], and the EggNog 4.5 database[19]. Secondary

metabolite gene clusters were predicted using antiSMASH[20]. Secreted proteins were predicted using SignalP v4.1[21] and Phobius v1.01[22]. Functional annotation and EVidenceModeler gene models were converted into GenBank format using GAG and tbl2asn. Orthologous proteins were identified using ProteinOrtho5[23]. Phylogeny was inferred by randomly selecting 500 orthologous protein groups corresponding to BUSCO[12] conserved single copy orthologs, concatenated for each genome, aligned using MAFFT v7.221[24], and analyzed in RAxML v8.2.8[25] (GTR substitution model, 1,000 bootstrap replicates) using *Botrytis cinerea* as an outgroup. Gene ontology (GO terms) functional enrichment analysis was run using GOATOOLS v0.6.10 (https://github.com/tanghaibao/goatools) using a p-value threshold of 0.001 (go.obo format 1.2 release 2016-12-02). Python wrapper scripts to run the pipeline described herein are part of a package called funannotate and is publically available at https://github.com/nextgenusfs/funannotate.

Supplementary Tables

Supplementary Table 1. Fungal isolates used in this study.[1]

Species	Isolate	BioSample Accession	Genome Accession	Isolate location	Date of collection	Reference
P. destructans	20631-21	SAMN03382554	LAJJ00000000	*Myotis lucifugus*, New York, USA	2008	[26]
P. verrucosus	UAMH10579	SAMN03382556	LAJO00000000	Sphagnum peat, Alberta, Canada	2002	This study
Pseudogymnoascus sp.	03VT05	SAMN03382557	LAJN00000000	Hibernacula soil, Vermont, USA	2008	This study
Pseudogymnoascus sp.	05NY08	SAMN03382558	LAJM00000000	Hibernacula soil, New York, USA	2008	This study
Pseudogymnoascus sp.	24MN13	SAMN03382560	LAJK00000000	Hibernacula soil, Minnesota, USA	2008	This study
Pseudogymnoascus sp.	WSF3629	SAMN03382555	LAJI00000000	Amorophous peat, Wisconsin, USA	1960	This study
Pseudogymnoascus sp.	23342-1-I1	SAMN03382559	LAJL00000000	*Perimyotis subflavus*, Wisconsin, USA	2008	This study
P. destructans	CCF3941	SAMN04883151	-	*Myotis myotis*, Mala Amerika, Czech Republic	2010	[27]
P. destructans	CCF3942	SAMN04883152	-	*Myotis myotis*, Mala Amerika, Czech Republic	2010	[27]
P. destructans	CCF4124	SAMN04883153	-	*Myotis myotis*, Horni Alberice, Czech Republic	2011	[28]
P. destructans	CCF4125	SAMN04883154	-	*Myotis myotis*, Horni Alberice, Czech Republic	2011	[28]

[1] Fungal strains are deposited in the Center for Forest Mycology Research Culture Collection (US Forest Service, Madison, WI) and are available upon request.

Supplementary Table 2. Genomes used for maximum likelihood phylogeny and estimation of evolutionary divergence.

Species Name	Strain	NCBI Accession
Aspergillus nidulans	FGSC A4	GCF_000149205.1
Botrytis cinerea	B05.10	GCF_000143535.1
Fusarium fujikuroi	IMI 58289	GCA_900079805.1
Neurospora crassa	OR74A	GCA_000182925.2
Penicillium chrysogenum	P2niaD18	GCA_000710275.1
Pseudogymnoascus destructans	20631-21	GCA_001641265.1
Pseudogymnoascus "pannorum var. pannorum"	ATCC 16222	GCA_001630605.1
Pseudogymnoascus "pannorum var. pannorum"	M1372	GCA_000497305.1
Pseudogymnoascus sp. 03VT05	03VT05	GCA_001662645.1
Pseudogymnoascus sp. 04NY16	04NY16	GCA_001630575.1
Pseudogymnoascus sp. 05NY08	05NY08	GCA_001662605.1
Pseudogymnoascus sp. 23342-1-I1	23342-1-I1	GCA_001662575.1
Pseudogymnoascus sp. 24MN13	24MN13	GCA_001662595.1
Pseudogymnoascus sp. BL308	BL308	GCA_001630595.1
Pseudogymnoascus sp. BL549	BL549	GCA_001630585.1
Pseudogymnoascus sp. VKM F-103	VKM F-103	GCA_000750895.1
Pseudogymnoascus sp. VKM F-3557	VKM F-3557	GCA_000750665.1
Pseudogymnoascus sp. VKM F-3775	VKM F-3775	GCA_000750715.1
Pseudogymnoascus sp. VKM F-3808	VKM F-3808	GCA_000750675.1
Pseudogymnoascus sp. VKM F-4246	VKM F-4246	GCA_000750735.1
Pseudogymnoascus sp. VKM F-4281 (FW-2241)	VKM F-4281 (FW-2241)	GCA_000750745.1
Pseudogymnoascus sp. VKM F-4513 (FW-928)	VKM F-4513 (FW-928)	GCA_000750755.1
Pseudogymnoascus sp. VKM F-4514 (FW-929)	VKM F-4514 (FW-929)	GCA_000750795.1
Pseudogymnoascus sp. VKM F-4515 (FW-2607)	VKM F-4515 (FW-2607)	GCA_000750805.1
Pseudogymnoascus sp. VKM F-4516 (FW-969)	VKM F-4516 (FW-969)	GCA_000750815.1
Pseudogymnoascus sp. VKM F-4517 (FW-2822)	VKM F-4517 (FW-2822)	GCA_000750875.1
Pseudogymnoascus sp. VKM F-4518 (FW-2643)	VKM F-4518 (FW-2643)	GCA_000750925.1
Pseudogymnoascus sp. VKM F-4519 (FW-2642)	VKM F-4519 (FW-2642)	GCA_000750935.1
Pseudogymnoascus sp. VKM F-4520 (FW-2644)	VKM F-4520 (FW-2644)	GCA_000750995.1
Pseudogymnoascus sp. WSF 3629	WSF 3629	GCA_001662585.1
Pseudogymnoascus verrucosus	UAMH 10579	GCF_001662655.1
Sclerotinia sclerotiorum	1980 UF-70	GCF_000146945.1

Supplementary Table 3. Gene ontology enrichment of biological processes in *P. destructans*

GO Term	enrichment	Description	Pd Ratio	Pop Ratio	Pd Count	FDR P-value
**GO:0009987**	over	cellular process	1670/4707	12725/40899	1670	0
**GO:0044237**	over	cellular metabolic process	1338/4707	10412/40899	1338	0.004
**GO:0055085**	under	transmembrane transport	300/4707	3582/40899	300	0
**GO:0005975**	under	carbohydrate metabolic process	182/4707	2268/40899	182	0
**GO:0008150**	under	biological_process	3104/4707	28366/40899	3104	0
**GO:0055114**	under	oxidation-reduction process	438/4707	4739/40899	438	0
**GO:0006351**	under	transcription, DNA-templated	88/4707	1216/40899	88	0.002
**GO:0097659**	under	nucleic acid-templated transcription	88/4707	1216/40899	88	0.002
**GO:0008152**	under	metabolic process	2125/4707	19663/40899	2125	0.022
**GO:0032774**	under	RNA biosynthetic process	114/4707	1397/40899	114	0.032

Supplementary Table 4. Secondary metabolite backbone enzymes found in each genome using antiSMASH.

	P. sp. 23342-1-I1	P. sp. WSF3629	P. sp. 24MN13	P. sp. 05NY08	P. sp. 03VT05	P. verrucosus	P. destructans
**NRPS**	8	9	6	5	4	6	3
**PKS**	15	12	8	8	5	11	7
**HYBRID**	1	4	3	2	1	2	0
**OTHER**	12	10	8	7	9	8	4
**TOTAL**	36	35	25	22	19	27	14

Supplementary Figures

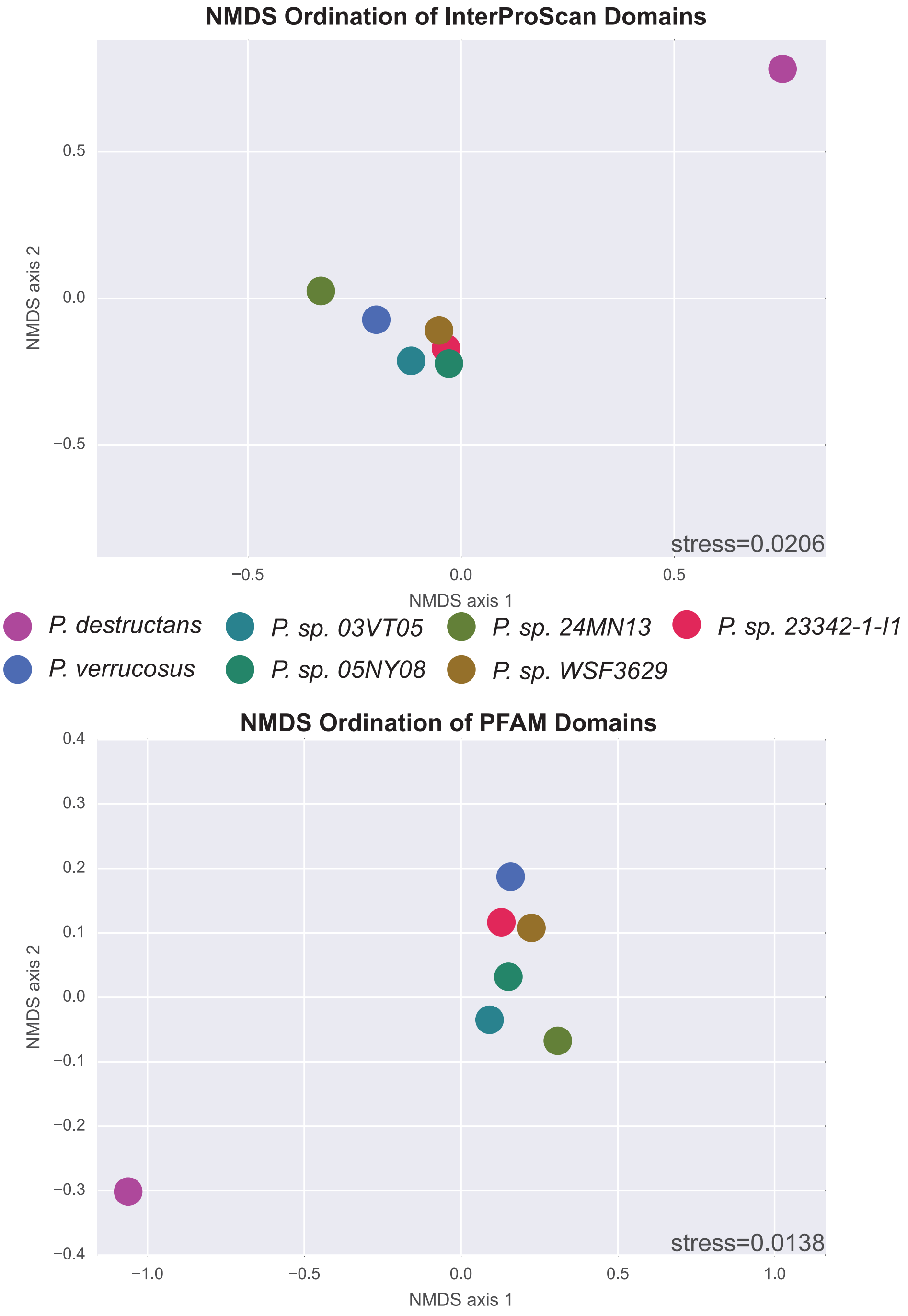

Supplementary Figure 1. Visual depiction using a NMDS ordination of InterProScan and Pfam protein counts per genome.

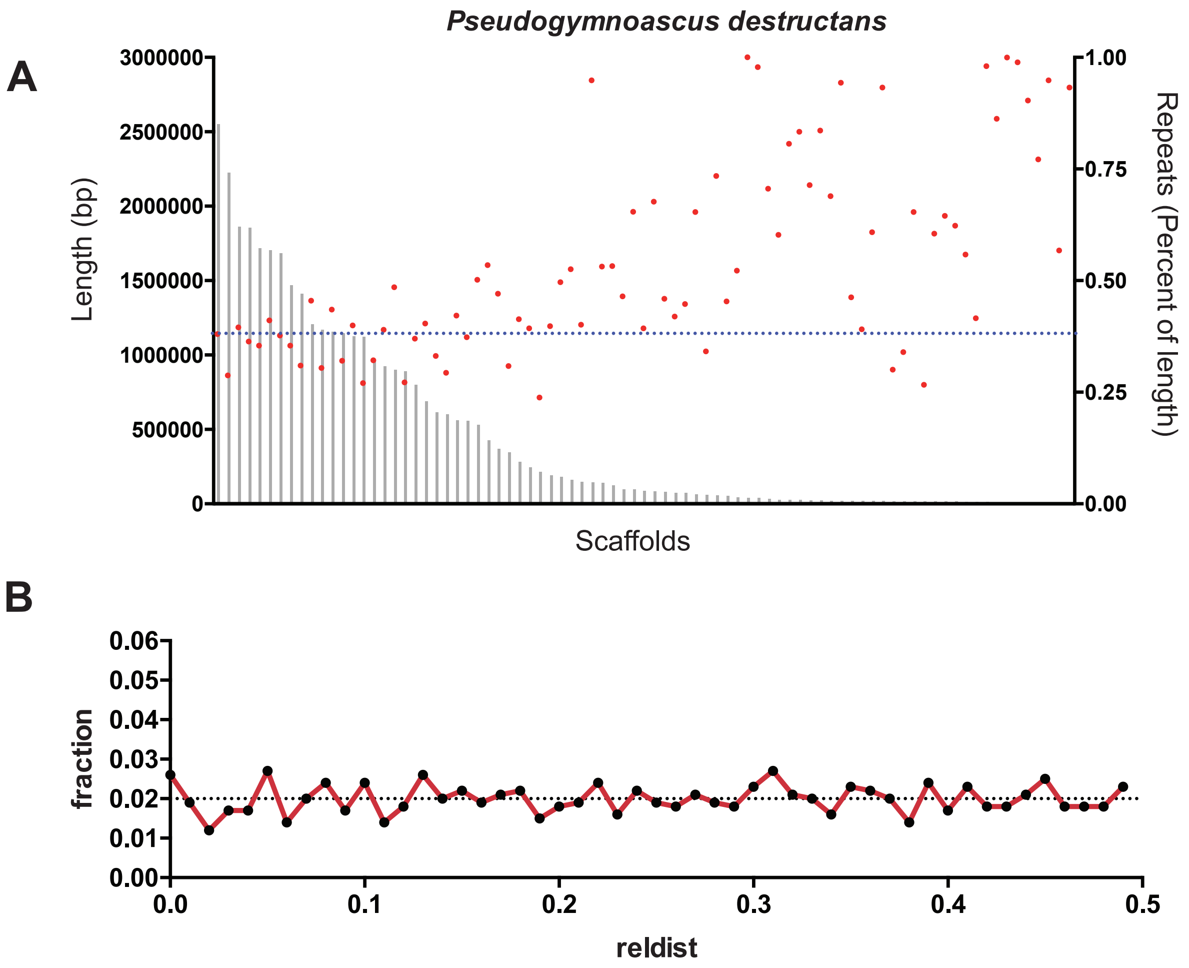

Supplementary Figure 2. (A) Distribution of repetitive DNA sequences per scaffold in *Pseudogymnoascus destructans*. The genome assembly contains 38.17% repetitive DNA sequences (denoted by the blue dashed line). Each scaffold contains a considerable amount of repetitive DNA indicating a somewhat uniform distribution of repeats across the genome. (B) We calculated the relative distance (jaccard intersection using bedtools[29]) of lineage specific (unique) genes compared to repetitive DNA elements (expanded by 1 kb on both sides of the repetitive element). Fractions large than 0.5 would indicate non-uniform distribution. For *P. destructans* the intersection of lineage specific genes with repetitive elements is less than 0.03 and therefore uniformly distributed.

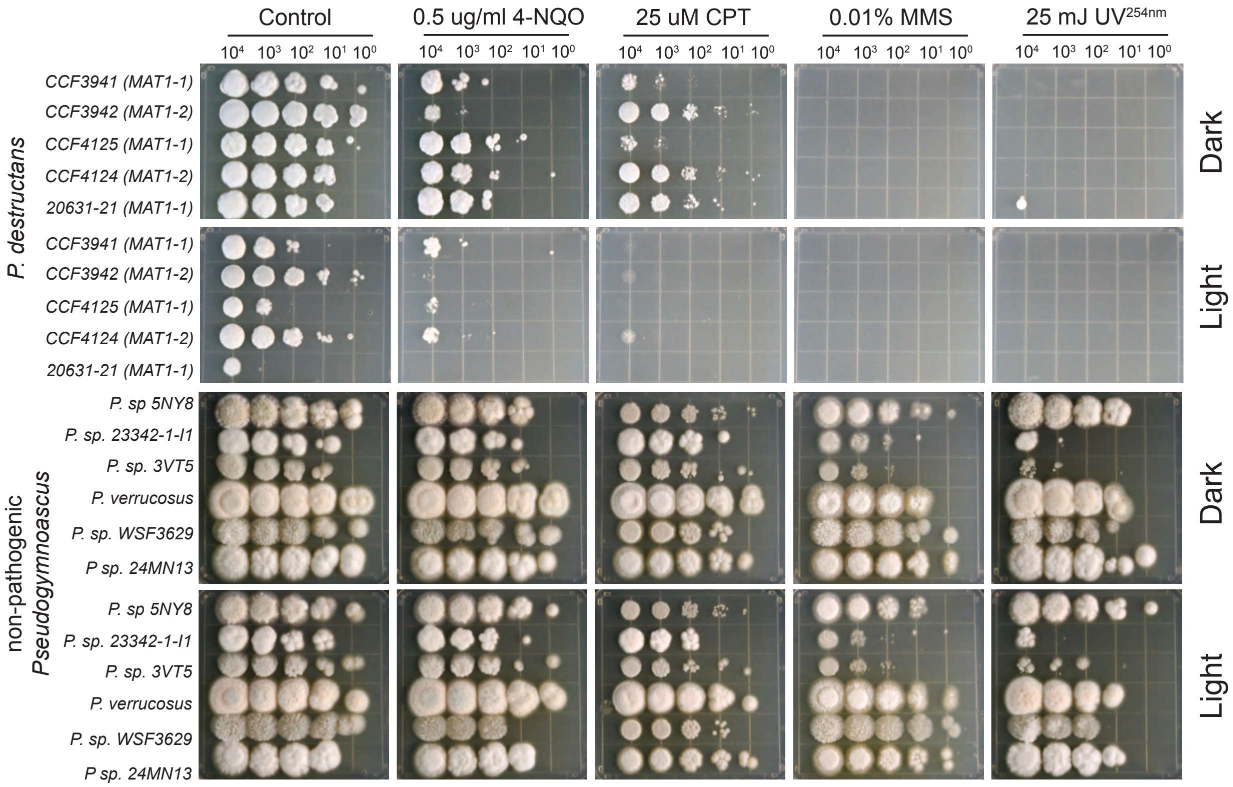

Supplementary Figure 3. Incubation in light is insufficient to repair DNA lesions in *P. destructans*. Fungal spores of five isolates of *P. destructans* were serially diluted, inoculated on appropriate medium, and growth was quantified after 7-day incubation at 15ºC. To test if white light increased survival (photoreactivation) after treatment with DNA damaging agent, plates were incubated in light and compared to the control plate incubated in the dark. Growth of *P. destructans* isolates in white light is inhibited compared to incubation in dark. A subset of this experiment is presented in Figure 4.

Supplementary References

1 Smit, A. & Hubley, R. *RepeatModeler Open-1.0*, <http://www.repeatmasker.org/> (2015).

2 Smit, A., Hubley, R. & Green, P. *RepeatMasker Open-4.0*, <http://www.repeatmasker.org/> (2015).

3 Grabherr, M. G. *et al.* Full-length transcriptome assembly from RNA-Seq data without a reference genome. *Nat. Biotechnol.* **29**, 644-652, doi:10.1038/nbt.1883 (2011).

4 Kim, D., Langmead, B. & Salzberg, S. L. HISAT: a fast spliced aligner with low memory requirements. *Nat. Methods* **12**, 357-360, doi:10.1038/nmeth.3317 (2015).

5 Haas, B. J. *et al.* Automated eukaryotic gene structure annotation using EVidenceModeler and the Program to Assemble Spliced Alignments. *Genome Biol.* **9**, R7, doi:10.1186/gb-2008-9-1-r7 (2008).

6 Stanke, M. & Morgenstern, B. AUGUSTUS: a web server for gene prediction in eukaryotes that allows user-defined constraints. *Nucleic Acids Res.* **33**, W465-467, doi:10.1093/nar/gki458 (2005).

7 Lomsadze, A., Burns, P. D. & Borodovsky, M. Integration of mapped RNA-Seq reads into automatic training of eukaryotic gene finding algorithm. *Nucleic Acids Res.* **42**, e119, doi:10.1093/nar/gku557 (2014).

8 Hoff, K. J., Lange, S., Lomsadze, A., Borodovsky, M. & Stanke, M. BRAKER1: Unsupervised RNA-Seq-Based Genome Annotation with GeneMark-ET and AUGUSTUS. *Bioinformatics* **32**, 767-769, doi:10.1093/bioinformatics/btv661 (2016).

9 Slater, G. S. C. & Birney, E. Automated generation of heuristics for biological sequence comparison. *BMC Bioinformatics* **6**, 31, doi:10.1186/1471-2105-6-31 (2005).

10 Wu, T. D., Reeder, J., Lawrence, M., Becker, G. & Brauer, M. J. GMAP and GSNAP for Genomic Sequence Alignment: Enhancements to Speed, Accuracy, and Functionality. *Methods Mol. Biol.* **1418**, 283-334, doi:10.1007/978-1-4939-3578-9_15 (2016).

11 Lowe, T. M. & Eddy, S. R. tRNAscan-SE: a program for improved detection of transfer RNA genes in genomic sequence. *Nucleic Acids Res.* **25**, 955-964 (1997).

12 Simão, F. A., Waterhouse, R. M., Ioannidis, P., Kriventseva, E. V. & Zdobnov, E. M. BUSCO: assessing genome assembly and annotation completeness with single-copy orthologs. *Bioinformatics* **31**, 3210-3212, doi:papers2://publication/doi/10.1093/bioinformatics/btv351 (2015).

13 Camacho, C. *et al.* BLAST+: architecture and applications. *BMC Bioinformatics* **10**, 421, doi:10.1186/1471-2105-10-421 (2009).

14 Finn, R. D. *et al.* The Pfam protein families database: towards a more sustainable future. *Nucleic Acids Res.* **44**, D279-285, doi:10.1093/nar/gkv1344 (2016).

15 Jones, P. *et al.* InterProScan 5: genome-scale protein function classification. *Bioinformatics* **30**, 1236-1240, doi:10.1093/bioinformatics/btu031 (2014).

16 Rawlings, N. D., Waller, M., Barrett, A. J. & Bateman, A. MEROPS: the database of proteolytic enzymes, their substrates and inhibitors. *Nucleic Acids Res.* **42**, D503-509, doi:10.1093/nar/gkt953 (2014).

17 Yin, Y. *et al.* dbCAN: a web resource for automated carbohydrate-active enzyme annotation. *Nucleic Acids Res.* **40**, W445-451, doi:10.1093/nar/gks479 (2012).

18 The UniProt Consortium. UniProt: the universal protein knowledgebase. *Nucleic Acids Res.* **45**, D158-D169, doi:10.1093/nar/gkw1099 (2017).

19 Huerta-Cepas, J. *et al.* eggNOG 4.5: a hierarchical orthology framework with improved functional annotations for eukaryotic, prokaryotic and viral sequences. *Nucleic Acids Res.* **44**, D286-293, doi:10.1093/nar/gkv1248 (2016).
20 Weber, T. *et al.* antiSMASH 3.0-a comprehensive resource for the genome mining of biosynthetic gene clusters. *Nucleic Acids Res.* **43**, W237-243, doi:10.1093/nar/gkv437 (2015).
21 Petersen, T. N., Brunak, S., von Heijne, G. & Nielsen, H. SignalP 4.0: discriminating signal peptides from transmembrane regions. *Nat. Methods* **8**, 785-786, doi:10.1038/nmeth.1701 (2011).
22 Käll, L., Krogh, A. & Sonnhammer, E. L. L. A combined transmembrane topology and signal peptide prediction method. *J. Mol. Biol.* **338**, 1027-1036, doi:10.1016/j.jmb.2004.03.016 (2004).
23 Lechner, M. *et al.* Orthology detection combining clustering and synteny for very large datasets. *PLoS One* **9**, e105015, doi:10.1371/journal.pone.0105015 (2014).
24 Katoh, K., Misawa, K., Kuma, K.-i. & Miyata, T. MAFFT: a novel method for rapid multiple sequence alignment based on fast Fourier transform. *Nucleic Acids Res.* **30**, 3059-3066 (2002).
25 Stamatakis, A. RAxML version 8: a tool for phylogenetic analysis and post-analysis of large phylogenies. *Bioinformatics* **30**, 1312-1313, doi:10.1093/bioinformatics/btu033 (2014).
26 Drees, K. P. *et al.* Use of multiple sequencing technologies to produce a high-quality genome of the fungus *Pseudogymnoascus destructans*, the causative agent of bat white-nose syndrome. *Genome announcements* **4**, doi:10.1128/genomeA.00445-16 (2016).
27 Kubátová, A., Koukol, O. & Novakova, A. *Geomyces destructans*, phenotypic features of some Czech isolates. *Czech Mycology* **63**, 65-75 (2011).
28 Palmer, J. M. *et al.* Molecular characterization of a heterothallic mating system in *Pseudogymnoascus destructans*, the fungus causing white-nose syndrome of bats. *G3* **4**, 1755-1763, doi:10.1534/g3.114.012641 (2014).
29 Quinlan, A. R. & Hall, I. M. BEDTools: a flexible suite of utilities for comparing genomic features. *Bioinformatics* **26**, 841-842, doi:10.1093/bioinformatics/btq033 (2010).
