# Supplementary material for: Extreme sensitivity to ultraviolet light in the fungal pathogen causing white-nose syndrome of bats
Source: Nat Commun. 2018 Jan 2;9:35. doi: 10.1038/s41467-017-02441-z (PMC5750222; doi:10.1038/s41467-017-02441-z)
Supplement: Supplementary file 3 — Description of Additional Supplementary Files [file 41467_2017_2441_MOESM3_ESM.pdf]

Description of Additional Supplementary Files

File Name: Supplementary Data 1
Description: Genome annotations for Pseudogymnoascus destructans 20631-21. [Excel file]

File Name: Supplementary Data 2
Description: Genome annotations for Pseudogymnoascus sp. WSF 3629. [Excel File]

File Name: Supplementary Data 3
Description: Genome annotations for Pseudogymnoascus verrucosus UAMH10579. [Excel File]

File Name: Supplementary Data 4
Description: Genome annotations for Pseudogymnoascus sp. 23342-1-I1. [Excel File]

File Name: Supplementary Data 5
Description: Genome annotations for Pseudogymnoascus sp. 05NY08. [Excel File]

File Name: Supplementary Data 6
Description: Genome annotations for Pseudogymnoascus sp. 03VT05. [Excel File]

File Name: Supplementary Data 7
Description: Genome annotations for Pseudogymnoascus sp. 24MN13. [Excel File]

File Name: Supplementary Data 8
Description: NEXUS file for generating fossil-calibrated phylogenetic tree using r8s. [NEXUS format]

File Name: Supplementary Data 9
Description: Orthologous protein groups identified from 7 Pseudogymnoascus genomes using ProteinOrtho5. [Excel File]

File Name: Supplementary Data 10
Description: Carbohydrate activating enzyme (CAZymes) counts per genome. [Excel File]

File Name: Supplementary Data 11
Description: Biolog phenotypic microarray assessment of ability to grow on 190 different carbon sources. [Excel File]

File Name: Supplementary Data 12
Description: MEROPS proteases identified in the Pseudogymnoascus genomes. [Excel File]

File Name: Supplementary Data 13
Description: InterProScan domains identified in the Pseudogymnoascus genomes. [Excel File]

File Name: Supplementary Data 14
Description: Pfam domains identified in the Pseudogymnoascus genomes. [Excel File]

File Name: Supplementary Data 15
Description: Secondary metabolite gene clusters in Pseudogymnoascus destructans. [Excel File]

File Name: Supplementary Data 16
Description: Secondary metabolite gene clusters in Pseudogymnoascus sp. WSF 3629. [Excel File]

File Name: Supplementary Data 17
Description: Secondary metabolite gene clusters in Pseudogymnoascus verrucosus UAMH10579. [Excel File]

File Name: Supplementary Data 18
Description: Secondary metabolite gene clusters in Pseudogymnoascus sp. 23342-1-I1. [Excel File]

File Name: Supplementary Data 19
Description: Secondary metabolite gene clusters in Pseudogymnoascus sp. 05NY08. [Excel File]

File Name: Supplementary Data 20
Description: Secondary metabolite gene clusters in Pseudogymnoascus sp. 03VT05. [Excel File]

File Name: Supplementary Data 21
Description: Secondary metabolite gene clusters in Pseudogymnoascus sp. 24MN13. [Excel File]

File Name: Supplementary Data 22
Description: Summary of BLAST search results for DNA repair machinery in Pseudogymnoascus genomes as well as Botrytis cinerea. [Excel File]
